# Gastrointestinal Elimination of Perfluorinated Compounds Using Cholestyramine and *Chlorella pyrenoidosa*

**DOI:** 10.1155/2013/657849

**Published:** 2013-09-09

**Authors:** Stephen J. Genuis, Luke Curtis, Detlef Birkholz

**Affiliations:** ^1^Faculty of Medicine, University of Alberta, 2935-66 Street, Edmonton, AB, Canada T6K 4C1; ^2^Forest Hills, NY 11375, USA; ^3^ALS Laboratory Group, University of Alberta and Environmental Division, Edmonton, AB, Canada T6E 5C1

## Abstract

*Background*. While perfluorinated compounds (PFCs) are a family of commonly used synthetic compounds with many applications, some PFCs remain persistent within the human body due, in part, to enterohepatic recirculation and renal tubular reabsorption. With increasing recognition of potential harm to human health associated with PFC bioaccumulation, interventions to facilitate elimination of these toxicants are welcome in order to potentially preclude or overcome illness. Minimal research has been undertaken thus far on methods to accelerate human clearance of PFCs. *Methods*. To test for possible oral treatments to hasten PFC elimination, a group of individuals with elevated PFC levels was treated with cholestyramine (CSM) and, after a break, was subsequently treated with *Chlorella pyrenoidosa* (CP). Stool samples were collected from all participants (i) prior to any treatment, (ii) during treatment with CSM, and (iii) during treatment with CP. *Results*. With CSM treatment, significant levels of three distinct PFCs were found in all stools, while levels were mostly undetectable prior to treatment. Following treatment with oral CP, undetectable or very low levels of all PFCs were noted in each sample tested. *Conclusion*. CSM appears to facilitate elimination of some common PFCs and may have some role in the clinical management of patients with accrued PFCs.

## 1. Introduction and Background

Perfluorinated compounds (PFCs) are a group of anthropogenic chemicals with many useful applications, including repelling stains on furniture/carpets/clothing as well as securing nonadherence in food packaging and nonstick cooking surfaces. Structurally, these compounds consist of a linear or branched carbon backbone, that is, entirely substituted by strong bonds fluorine atoms. The fluorine component of PFCs provides extremely low surface tension and accounts for their unique hydrophobic (water repelling) and lipophobic (lipid repelling) natures [1]. These compounds differ markedly from most other chemical surfactants in that they are very stable, nonreactive, and effective at low concentrations. With these unique properties, selected PFCs have been used to make commercial products that are resistant to both water and oil, are stain resistant, and can also withstand the extremes of temperature, pH, and oxidizing conditions.

### 1.1. Human PFC Exposure

With a number of useful applications, PFC compounds have come into common use in western culture. Human exposure in domestic and commercial settings has occurred as a result of processes such as inhalation of contaminated air, ingestion of tainted dust and foodstuffs, and dermal absorption in treated clothing. With inherent chemical stability and slow elimination from the human body, accrual of these persistent compounds continues in the population at large [2]. In addition, spills of PFCs have occurred where individuals have been exposed to elevated levels of these agents through avenues such as the water supply [3]. Furthermore, high levels of PFC exposures have been reported in drinking water sources located near plants manufacturing PFCs [4].

Relatively high levels of PFCs have also been found in the Greenlandic Inuit and other circumpolar populations who consume large quantities of fish and marine mammals contaminated with PFCs [5]. In the general western population, it is estimated that diet provides most (~91% Perfluorooctane sulfonate (PFOS); ~99% Perfluorooctanoic acid (PFOA)) of the total PFC intake. In some groups, however, drinking water as well as indoor air and house dust can also provide significant PFC exposure [6]. Human studies have also reported that PFCs can cross the placenta and are present in breast milk [7, 8]. One study reported significant declines in serum PFOS in breastfeeding mothers [8], presumably due to vertical transmission of some PFCs into the infant via breast milk.

Some human studies have reported significantly higher mean levels of serum PFC in males versus females (NHANES) [2]. The precise reason for the variance has not been confirmed but may be due, in part, to PFC losses with regular menstrual bleeding and through breast feeding. These routes of bioelimination likely account for the finding that, in postmenopausal women, a Japanese study reported that serum levels of PFOA and PFOS were higher compared to menstruating premenopausal women [9].

### 1.2. PFC Persistence in Humans

Some of the various subtypes within the PFC family include (i) PFHxS (perfluorohexane sulfonate), (ii) PFOS (perflurooctane sulfonate), (iii) PFOA (perfluorooctanoic acid) and (iv) PFNA (perfluorononanoic acid). Each of the individual PFCs has unique properties pertaining to toxicokinetics and bio-elimination. In general, PFCs are eliminated very slowly in humans. Various human studies have estimated plasma half-lives of PFCs to be about 8.5 to 8.8 years for PFHxS (range 2.8–27 years), 5.4 years for PFOS (range 2.4 to 21.7 years), and 2.3 to 3.8 years for PFOA (range 1.5 to 9.1 years) [10, 11].

Unlike fat-soluble lipophilic compounds such as organochlorine pesticides and polychorinated biphenyls (PCBs) which tend to accumulate in adipose tissue, perfluorinated compounds tend to persist in bile as well as in lean human tissue including muscle, liver, and kidney [12]. Much of the total body burden of PFCs remains in blood bound to plasma proteins [12].

### 1.3. Emerging PFC Health Concerns

Recent research has uncovered assorted health concerns related to exposure and accrual of PFCs, including issues related to gestational and prenatal contamination. In addition, animal and human studies have now linked PFC exposure with developmental toxicity, neurotoxicity, hepatotoxicity, carcinogenicity, metabolic dysregulation, immunotoxicity, and endocrine disruption.

High serum PFC levels in women, for example, have been linked to significantly lower fertility rates in a large Norwegian study [13, 14] and to significantly lower birth weights in a cohort of pregnant Danish women [15]. A study of 158 pregnant Chinese women reported that higher serum levels of PFOA were associated with significantly shorter gestation, lower birth weights, and lower 5-minute Apgar scores as compared to pregnant women with lower serum PFOA [16]. A recent case-control study reported the finding of significantly higher PFOA and PFOS serum levels in women with surgically treated endometriosis as compared to matched control women [17]. Studies examining associations between serum PFC levels and human semen studies have been inconsistent, with some studies reporting significantly lower levels of morphologically normal sperm cells in men with higher serum PFC levels and other studies showing little relationship [18]. A study of 169 young Danish men (aged 19 to 21 years) from a pregnancy cohort reported that higher in-utero exposure to PFOA (as measured by maternal serum samples at the 30th week of pregnancy) was associated with significantly lower sperm counts and significantly higher levels of luteinizing hormone and follicle-stimulating hormone [19]. Exposure to certain PFCs has also been linked to reproductive and developmental problems in animal studies [1]. 

Exposure to perfluorinated compounds may be associated with adverse cardiovascular outcomes and dysregulation of body weight. A confounder adjusted study of 1,216 US adults reported significantly higher levels of cardiovascular disease (including heart diseases, stroke and peripheral vascular disease) in subjects with higher serum PFOA concentrations [20]. A study of 664 young subjects (aged 12 to 30 years) reported that higher serum levels of PFOS were associated with significantly thickened carotid artery intima-media thickness (CIM) [21]. A study with lab mice reported that low-level oral PFOA exposure was associated with significantly higher body weights and significantly higher levels of leptin and insulin as compared to unexposed mice [22]. More recently, newly published research has suggested a link between human serum concentrations of PFOS, and PFOA and the development of osteoarthritis [23].

Several animal studies have reported that higher exposure to PFCs is associated with neurotoxicity [24]. A study of 10,546 children in the Ohio Valley reported that higher serum levels of PFHxS were associated with significantly higher risk of attention deficit hyperactivity disorder (ADHD), while higher serum levels of PFOA, PFOS or PFNA were not associated with higher rates of ADHD [25]. Another study of 571 children aged 12 to 15 years reported that higher serum levels of PFOA, PFOA, and PFHxS were all individually associated with significantly higher rates of ADHD [26]. A cross-sectional study of 1,766 US adults aged 60 to 85 years reported that higher levels of PFOS, PFOA, PFNA and PFHxS were associated with modest yet nonsignificantly lower rates of cognitive limitation [27].

A recent case-control study among the Greenlandic Inuit reported that higher serum PFC levels were linked to significantly higher risk of breast cancer [1]. Studies on chemical industry workers have reported that occupational exposure to PFOA has been linked to significantly higher levels of prostate cancer mortality and occupational exposure to PFOS is linked to significantly higher rates of bladder cancer mortality [24]. Several studies with experimental animals have reported that exposures to PFCs are associated with hepatotoxicity and cancers of the liver, breast, and thyroid [1]. 

A number of experimental studies have reported that bioaccumulation of PFCs at levels comparable to highly exposed humans can reduce immune function in rats and mice [28]. A study of 587 Faroe Islands children vaccinated with diphtheria and tetanus toxoids reported that higher serum levels of PFOS and PFOA were associated with significantly lower levels of tetanus and diphtheria antibody concentrations at 5 or 7 years. Levels of PFCs were relatively high in this circumpolar population due to consumption of large amounts of fish and marine mammals [29]. 

Furthermore, a recent paper discusses the impact of PFCs on the functioning of sex hormone receptors. PFHxS, PFOS, and PFOA all significantly impact estrogen receptors, and antagonize androgen receptors in a concentration-dependent manner [30]. Accordingly, it is important to consider the combined action, rather than just the impact of individual PFCs. 

### 1.4. Therapeutic Elimination of PFCs

While there has been little attention in scientific research to clinical detoxification [31], there is a clear need to develop methods to reduce the body burdens of PFCs and other persistent toxicants in order to preclude adverse outcomes. There has been minimal study on means to specifically facilitate removal of PFCs from the human body, and recent research exploring the potential elimination of these compounds through skin depuration demonstrated a lack of success [32]. Another potential approach is to give oral agents which reduce PFC absorption in the GI tract and/or increase toxicant excretion via blocking of reabsorption of toxics in the enterohepatic pathway. The most studied of these oral detoxifying agents has been the bile sequestering agent cholestyramine (CSM). Other agents proposed for oral detoxification of persistent toxics include clay and zeolite compounds, supplements of the algae *Chlorella pyrenoidosa *(CP), and vegetable fibers like Psyllium husks.

CSM and other bile acid sequestrants like colestipol and colesevelam are positively charged nondigestible resins that bind to enterohepatic bile acids in the intestines, forming an insoluble complex excreted in feces. CSM is primarily used for reducing serum LDL-cholesterol levels; various studies have reported that CSM lowers LDL-cholesterol levels by 12–20% [33]. Other studies have also suggested that CSM may have other useful clinical benefits including treatment of diabetes, treatment of pruritis associated with cholestatic disease and treatment of diarrhea occurring from bile acid malabsorption [33]. While CSM is generally a safe drug, it can cause side effects like bloating, constipation, abdominal pain, and reduced absorption of fat-soluble vitamins and some drugs [33]. 

CSM has also been hypothesized to reduce body burdens of fat-soluble toxins via the binding of lipophilic compounds to prevent reabsorption through the enterohepatic pathway, although published data is rather scarce. In 1984, the use of oral CSM in lab rats was reported to significantly increase levels of PFOA and PFOS in feces by about 10-fold [34]. A human case study was presented for 51-year-old scientific researcher whose body burden of PFCs was significantly reduced after taking 4 grams of CSM 3 times a day for 28 weeks [35]. CSM has been used to significantly reduce body burdens of chlordecone (kepone) in humans [36]. 

CSM has also been demonstrated to reduce zearalenone (a mycotoxin produced mainly by the fungi *Fusarium*) absorption in mice exposed to feed contaminated with zearalenone [37]. An in vitro study reported that CSM was very effective in absorbing the mycotoxins (mold toxins) fumonisin and zearalenone but only slightly effective in absorbing the mycotoxins nivalenol and deoxynivalenol [38].

*Chlorella* is a fresh water algae which may have useful detoxifying properties. The use of oral supplements of *Chlorella pyrenoidosa* has been reported to significantly reduce dioxin levels in breast milk of 35 nursing women in Japan [39]. Lab studies have reported that the use of *Chlorella* supplements significantly reduced the half-life of chlordecone in chlordecone-poisoned rats and significantly reduced liver toxicity and cadmium-accumulation in cadmium poisoned rats [40, 41].

Other potential detoxification modalities remain untested in relation to PFC elimination. Oral clay has been reported to reduce body burdens of mycotoxins such as aflatoxins and fumonisins in lab animals and humans [42]. While clay compounds and zeolites have also been found to bind heavy metals and other toxins in water, soil, and other mediums [43], zeolites have thus far not been effective at removing PFCs [35]. As a significant proportion of the PFCs appear to persist in plasma bound to proteins, intermittent phlebotomy is currently being investigated as a means to rapidly diminish the body burden of PFCs. This latter approach may have a particular role in contaminated reproductive-aged women wishing to conceive in the near future but also keen to avoid transplacental PFC transfer with subsequent fetal exposure. In this paper, we are presenting a retrospective case series exploring potentially innocuous oral interventions to facilitate elimination of accrued PFCs. 

## 2. Methods

### 2.1. Participant Recruitment

As part of their clinical assessments, individuals presenting to an environmental health clinic in Edmonton, Alberta, Canada, are assessed for evidence of toxicant exposure and bioaccumulation of potentially toxic compounds. If individuals have a history of potential exposure, PFC testing is undertaken. If a patient is found to have bioaccumulated high PFC levels, PFC testing is also discussed and offered to family members who have sustained similar exposure. Eight individuals with elevated PFC burdens were thus identified—4 females and 4 males were included in the study. This recruitment was a selection of convenience as these individuals were found to have elevated levels and all were keen to participate. As part of the effort to diminish their PFC burden, two unrelated detoxification agents—CSM and CP—were offered as potential therapeutic clinical modalities to possibly facilitate elimination of their PFC burden It was difficult to anticipate efficacy of such interventions as PFCs tend to repel both lipophilic and hydrophilic compounds in their function as stain-resistant compounds.

All 8 individuals were completely informed of the available information in the literature about known risks of PFCs, the lack of known interventions to facilitate PFC removal, and the experience discussed in the literature in relation to CSM and Chlorella. After considering the potential health concerns associated with PFHxS, each patient was very keen to participate. There was a particular unease about potential neurotoxic effects as each of the women with elevated PFHxS indicated concern about the possibility of vertical transmission in future pregnancies [26, 44, 45]. Each participant tried CSM and subsequently tried CP as a means to potentially enhance PFC excretion. All of the patients expressed an ardent intent to subsequently use whichever agent appeared to be most efficacious at facilitating elimination of their accrued PFCs. Rather than a prospective clinical trial, this report details a retrospective study involving analysis of data found after clinical intervention undertaken with the full consent of each patient. Ethical approval was obtained from the Health Research Ethics Board at the University of Alberta.

### 2.2. Stool Collection

Stool collection was obtained from each participant prior to any intervention and analyzed for levels of 4 common PFCs: PFHxS, PFOA PFOS, and PFNA. Each participant was then treated with CSM (4 grams once per day for one week). A break of three weeks ensued and then each participant was subsequently treated with CP (9 grams three times per day for one week). Stool samples were collected from all 8 patients on three occasions: (i) before the start of any therapy, (ii) while taking CSM but after using it for at least 5 consecutive days, and (iii) while taking CP but after using it for at least 5 consecutive days. Serum PFC levels were assessed prior to and subsequent to the stool testing to confirm that each PFC compound found in stool was present in each patient's serum both prior to, and following the interventions.

Stool samples were collected in a 500 mL glass jar obtained from ALS Laboratories. Samples were provided directly into the glass jars. Each of the glass bottles used for sampling was provided by ALS Laboratories and had undergone extensive cleaning and rinsing. The containers were deemed appropriate for collection with negligible risk of contamination: laboratory-grade phosphate-free detergent wash, acid rinse, multiple hot and cold deionized water rinses, oven-dried, and capping and packing in quality controlled conditions. Samples were delivered to the physician who provided them to ALS Laboratories for testing.

### 2.3. Stool Analysis

For fecal PFC analysis, methanol along with a mixture of isotopically labeled PFCs was added to 1.0 g of freeze-dried stool. The mixture was vortexed, sonicated, and centrifuged. A known amount of the extract was collected and concentrated to 100 *μ*L on a nitrogen evaporator. 200 *μ*L of 90% 20 mM acetic acid/10% methanol and instrument performance internal standard were added to the vial. Analysis for each sample was performed by liquid chromatograph tandem mass spectrometry using multiple reaction monitoring. Liquid chromatograph tandem mass spectrometry has already been extensively used to measure PFCs in rat feces [46]. 

Detection limits were 0.5 ng/g for each analyte, based on the lowest standard in the standard curve. Procedural blanks were run with each set of samples, and blanks were always below 0.5 ng/g. As mentioned, the extracts were concentrated to 100 *μ*L. To secure precision, the laboratory staff added 100 *μ*L of methanol and carefully marked the meniscus. When the sample was concentrated, it was brought to the meniscus line previously marked in order to keep the volumes reproducible. The following isotopically labeled standards used were (i) ^13^C_4_-PFOS-sodium perfluoro-1-[1,2,3,4-^13^C_4_] octanesulfonate; (ii) ^13^C_4_-PFOA-perfluoro-n-[1,2,3,4-^13^C_4_] octanoic acid; (iii) ^13^C_5_-PFNA-perfluoro-n-[1,2,3,4,5-^13^C_5_] nonanoic acid; and (iv) ^13^C_2_-PFDA-perfluoro-n-[1,2-^13^C_2_] decanoic acid. All of the standard calibration curves were linear. The standard concentrations used were: (i) 0.5 ppb, (ii) 2.0 ppb, (iii) 10.0 ppb, (iv) 30.0 ppb, and (v) 50.0 ppb.

## 3. Results and Discussion

PFC results prior to and after treatment are given in Tables 1–3. Tables 1 and 2 provide (i) pre-treatment serum and stool PFC levels (ii) fecal PFC levels after treatment with oral CSM and (iii) fecal PFC levels after treatment with oral CP following an intervening 3-week break between treatments.  Table 1 reports on results for the 4 female participants; Table 2 reports on the same indices for the males.  Table 3 provides the mean levels of PFCs in serum and stool prior to treatment and corresponding mean fecal levels after CSM treatment.  Table 4 lists 50%, 75%, 90%, and 95% levels of serum PFCs reported in the National Health and Nutrition Examination Study [2]. Each table reports on 4 different PFCs, including, (i) PFHxS (perfluorohexane sulfonate), (ii) PFOS (perfluorooctane sulfonate), (iii) PFOA (perfluorooctanoic acid), and (iv) PFNA (perfluorononanoic acid).

As reported in Tables 1 and 2, all participants had elevated levels of PFHxS in serum prior to treatment, yet none were excreting noteworthy amounts in stool prior to any intervention. In fact, all 8 study subjects had PFHxS serum levels well above the 95% NHANES percentile. Mean levels of PFHxS were 48.4 ng/g in females and 50.8 ng/g in males, (Table 3), which are both well above the 8.2 ng/g female and 8.5 ng/g male 95% percentiles reported in the NHANES study (Table 4) [2]. The mean serum levels of PFOS for this population were also on the higher side at 26 ng/g for females and 29 ng/g for males, which approaches the 75% percentile in the NHANES study. PFOA levels averaged 2.7 ng/g for females and 3.8 ng/g for males in this study, both of which are below the 50th percentile of the NHANES group. PFNA levels averaged 0.34 ng/g for the female patients and 0.36 ng/g for the male patients, well below the mean NHANES PFNA values.

The reason why the study population has such high PFHxS levels relative to the NHANES population may be related to ongoing exposure to carpet repeatedly treated with stain-resistant treatment with in-floor radiant heating under the carpeted floor in each case. Beesoon et al. [47] reported on a Canadian family of 7 with high serum PFHxS levels (27.5–423 ng/mL) following several carpet applications of Scotchgard carpet protector.

Despite abundant PFCs in serum, none of the participants showed evidence of noteworthy gastrointestinal elimination of any of the PFCs tested prior to treatment, with all levels in fecal samples below or just above the detection limits. After treatment with CSM, significant levels of PFHxS were found in the stools of all 8 patients, with patient #2 having a stool concentration of 460 ng/g PFHxS. Significant post-CSM stool levels of PFOS and PFOA were also seen in most of the patients, while no detectable PFNA was seen in the stools of any of the patients. Perhaps the effectiveness of the CSM was limited by the low initial concentrations of PFNA. The effectiveness of PFC removal by CSM varied from patient to patient, but each showed evidence of noteworthy PFC elimination when treated with CSM.

**Figure 1:**
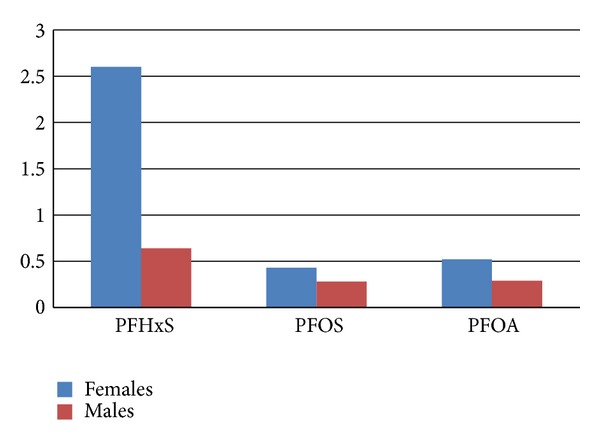
Ratio of PFCs in stool following CSM treatment versus pre-treatment serum.

Figure 1 compares post-CSM treatment mean stool levels of PFHxS, PFOS, and PFOA with pre-treatment mean serum levels of these 3 PFCs, separated by gender. The stool to serum ratio is highest in the case of PFHxS, especially in females, suggesting that perhaps CSM is especially effective at removing PFHxS. Another possibility is that the CSM may have been particularly effective in removing PFHxS due to the high body PFHxS stores in this group.

Table 1 reports that levels of fecal PFCs following *Chlorella pyrenoidosa* treatment were all below detection limit or just above detection limit for all 4 PFC compounds in all 8 patients. With no new PFC exposure, follow-up serum PFC testing was undertaken after the CP detoxification interventions were completed. Persistently high blood levels of PFCs in each patient confirmed that CP was ineffective despite the ongoing presence of abundant serum PFCs. Accordingly, *Chlorella pyrenoidosa *does not seem to be a clinically useful way of interrupting enterohepatic recycling of PFCs.

Despite the suggestive evidence that CSM is effective at facilitating the release of PFCs, this retrospective analysis has limitations. One limitation might involve the potential nonhomogeneity of stool samples, as PFC concentrations may vary from site to site within any given stool sample, which may alter numerical results. Furthermore, without precise knowledge of the preexisting body burden, it is not possible to determine the rate of decline by analyzing the concentration in stool. Thus, the total amount or rate of PFC excretion following CSM therapy is not quantified. 

Ongoing serum PFC levels for each patient were not presented in this report. Consecutive serum PFC levels taken over a short period of time do not correlate precisely with changes in body burden for several reasons. Toxicokinetics of most adverse chemical compounds are not completely understood and PFC levels within a given body compartment can fluctuate. Tissue and serum levels do not maintain a steady equilibrium, with a potential for rapid mobilization of toxicants from one compartment to another. Levels of some compounds within a single compartment can multiply quickly in response to various determinants potentially including fever, exercise, or a shift in caloric status [48], thus rendering changes in serum values unreliable as short term indicators of body burden. Secondly, there is a significant margin of error within parts per billion serum assessment, and a one-week use of any agent may not be adequate to manifest a sufficient decline in PFC body burden to be noted by serum testing. In spite of these limitations, however, there remains compelling and consistent evidence that CSM, but not CP, appears to facilitate PFC excretion for three of the 4 PFCs analyzed at the varying serum levels found in these individuals.

## 4. Conclusion

PFCs accumulate in human blood and tissues, have long elimination half-lives, and are increasingly linked to assorted health concerns. Recent evidence confirms that many people within the general population have had exposure to, and bioaccumulation of PFCs within their body. Interventions are needed to facilitate removal of PFCs in order to preclude the development of health sequelae related to accrual of these toxicant compounds, including outcomes resulting from gestational exposures in reproductive-aged women. Thus far, there is minimal research on interventions to facilitate elimination of these compounds. Although the scale of elimination in relation to the accumulated PFC body burden cannot be quantified by this preliminary study, these findings provide compelling evidence that oral cholestyramine is effective at hastening elimination of various PFCs from the human body, particularly PFHxS. Oral *Chlorella pyrenoidosa* was not found to be effective at removing PFCs. More studies are needed to determine ideal strategies and dosing regimens which utilize cholestyramine and/or other agents for enhancing the elimination of toxic PFCs.

## 5. Key Findings

- Cholestyramine (CSM) appears to be an effective agent for facilitating the gastrointestinal excretion of PFHxS, PFOA, and PFOS.
- *Chlorella pyrenoidosa* (CP) does not appear to be effective at enhancing the gastrointestinal excretion of perfluorinated compounds.

## Figures and Tables

**Table 1:** Removal of persistent perfluorinated compounds with cholestyramine (CSM) and *Chlorella  pyrenoidosa* (CP)—female subjects.* Objective*. To assess whether CSM is consistently effective at removing PFCs from 4 female patients with high PFC levels; posttreatment stool level: after daily CSM or after daily CP. Units—ng/g: blood and ng/g: stool.

Subject	Type of test	PFHxS	PFOS	PFOA	PFNA
#1 female 48	Serum	16.3**	5.67	1.27	0
	Pretreatment stool	1.0	<1.0	<1.0	<1.0
	Post-CSM stool	3.4	1.0	1.1	<1.0
	Post-CP stool	<1.0	<1.0	<1.0	<1.0

#2 female 24	Serum	35.8**	26.5	2.17	0
	Pretreatment stool	<1.0	<1.0	<1.0	<1.0
	Post-CSM stool	460	2.1	2.1	<1.0
	Post-CP stool	<1.0	<1.0	<1.0	<1.0

#3 female 22	Serum	44.1**	27.3	2.33	0.74
	Pretreatment stool	<1.0	1.4	<1.0	<1.0
	Post-CSM stool	16	5.3	<1.0	<1.0
	Post-Chlorella stool	<1.0	1.1	<1.0	<1.0

#4 female 20	Serum	97.5**	44.7	5.04	0.62
	Pretreatment stool	1.7	1.5	<1.0	<1.0
	Post-CSM stool	26.6	36.9	2	<1.0
	Post-CP stool	1.1	<1.0	<1.0	<1.0

**Denotes PFC serum value above 95th percentile, NHANES study.

**Table 2:** Removal of persistent perfluorinated compounds with cholestyramine and chlorella—male Subjects. *Objective*. To assess whether CSM is consistently effective at removing PFCs from 4 male patients with high PFC levels; posttreatment stool level: after daily CSM or after daily CP. Units—ng/g: blood and ng/g: stool.

Subject	Type of test	PFHxS	PFOS	PFOA	PFNA
#5 male 54	Serum	34.5**	8.58	1.97	0
	Pretreatment stool	<1.0	<1.0	<1.0	<1.0
	Post-CSM stool	59.4	4.1	<1.0	<1.0
	Post-CP stool	<1.0	<1.0	<1.0	<1.0

#6 male 25	Serum	30.3**	11.3	3.99	0.89
	Pretreatment stool	1	1.3	<1.0	1.1
	Post-CSM Stool	13.7	<1.0	1.4	<1.0
	Post-CP Stool	<1.0	<1.0	<1.0	<1.0

#7 male 19	Serum	92.1**	58.8	5.13	0.54
	Pretreatment stool	1.1	<1.0	<1.0	<1.0
	Post-CSM Stool	30.1	23.4	1.2	<1.0
	Post-CP Stool	<1.0	<1.0	<1.0	<1.0

#8 male 17	Serum	46.5**	37.5	3.99	0
	Pretreatment stool	1.8	<1.0	<1.0	<1.0
	Post-CSM Stool	24.7	4.5	1.3	<1.0
	Post-CP Stool	1.2	<1.0	<1.0	<1.0

**Denotes PFC serum value above 95th percentile, NHANES study.

**Table 3:** Mean levels of PFCs in serum prior to treatment and stool after CSM treatment.

PFC species	Pretreatment serum PFC species in ng/g Mean (SD)		Stool PFC post-CSM in ng/g Mean (SD)		Ratio: post-CSM stool: serum pre-treatment	
	Female	Male	Female	Male	Female	Male
PFHxS	48.4 (34.7)	50.8 (28.3)	126 (222)	32 (19.5)	2.60	0.63
PFOS	26 (23.7)	29 (23.7)	11.3 (17.1)	8.2 (10.2)	0.43	0.28
PFOA	2.7 (1.6)	3.8 (1.3)	1.4 (0.76)	1.1 (0.41)	0.52	0.29
PFNA	0.34 (0.40)	0.36 (0.44)	0.5*	0.5*	N/A	N/A

*All PFNA post-treatment stool levels below detection limit of 1 ng/g.

**Table 4:** NHANES serum levels—all ages—2009 to 2010 [2]. 2,094 total subjects including 1,041 females and 1,053 males; all levels in *µ*g/L, which are approximately equal to ng/g.

	PFHxS		PFOS		PFOA		PFNA	
	Female	Male	Female	Male	Female	Male	Female	Male
Geometric mean	1.72	2.17	18.4	23.2	3.50	4.47	0.861	1.09
50% percentile	1.60	2.10	18.2	23.9	3.60	4.60	0.900	1.10
75% percentile	2.90	3.40	27.4	32.2	5.20	6.30	1.30	1.60
90% percentile	5.80	6.10	39.8	45.3	7.10	8.40	2.20	2.40
95% percentile	8.20	8.50	46.6	62.7	8.60	10.7	3.00	4.00
